# Supplementary material for: Bayesian analysis of retinotopic maps
Source: eLife. 2018 Dec 6;7:e40224. doi: 10.7554/eLife.40224 (PMC6340702; doi:10.7554/eLife.40224)
Supplement: Supplementary file 1. — To evaluate the accuracy of the predictions of retinotopic maps, we employ a cross-validation schema. Each subject’s 12 retinotopic mapping scans were divided into one large set of validation data as well as 21 smaller sets of training data. An additional dataset of all 12 scans was used for analysis of retinotopic properties not linked to evaluation of the quality of the predicted maps. [file elife-40224-supp1.pdf]

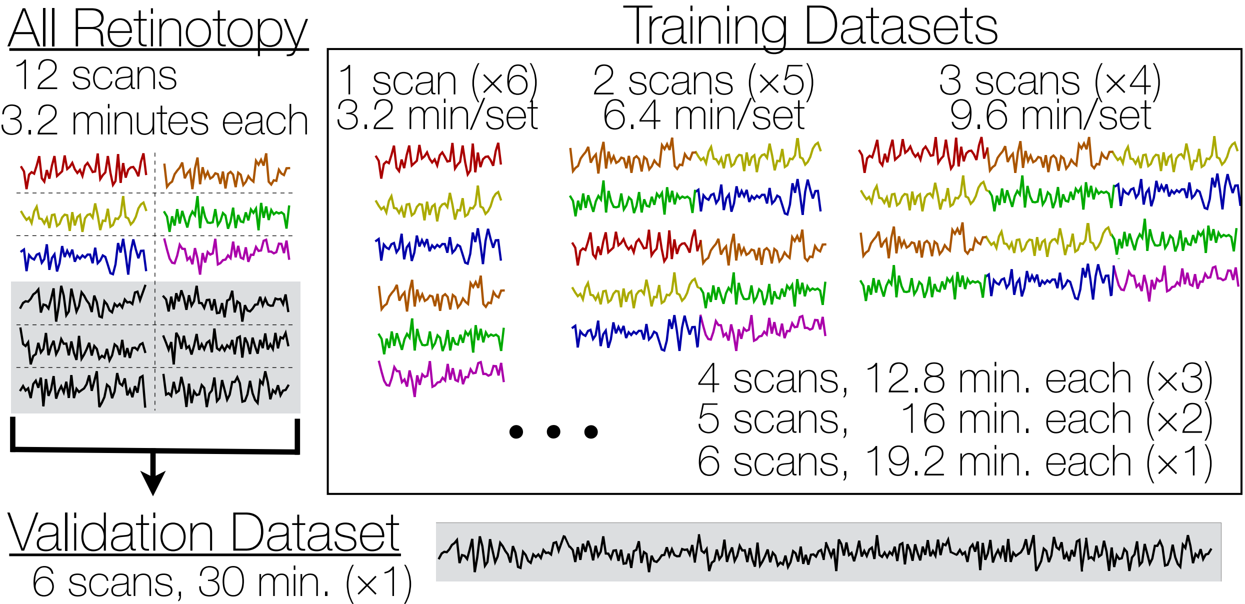
All Retinotopy
12 scans
3.2 minutes each
Training Datasets
1 scan (×6)
3.2 min/set
2 scans (×5)
6.4 min/set
3 scans (×4)
9.6 min/set
4 scans, 12.8 min. each (×3)
5 scans, 16 min. each (×2)
6 scans, 19.2 min. each (×1)
Validation Dataset
6 scans, 30 min. (×1)
