# Supplementary material for: Bayesian analysis of retinotopic maps
Source: eLife. 2018 Dec 6;7:e40224. doi: 10.7554/eLife.40224 (PMC6340702; doi:10.7554/eLife.40224)
Supplement: Supplementary file 2. — (A) The group-average polar angle (top) and eccentricity (bottom) maps. The cortical surface is inflated to a sphere then flattened to a map. (B) The model of retinotopy shown with polar angle plotted on the left and eccentricity plotted on the right hemispheres. (C) The retinotopic prior is constructed from the group-average data using an updated version of the method described by Benson et al. (2014). Note that while only eccentricity is shown, polar angle and eccentricity are registered simultaneously. The checkerboard underlay illustrates the anatomical warping. (D) There is approximate agreement between the boundaries of visual areas V1, V2, and V3 as defined by two atlases. The Wang et al. maximum probability atlas (2015) and the retinotopic template defined here have similar boundaries. The template extends from 0° to 90° eccentricity, whereas the Wang et al atlas is limited to the field of view of their experiments (14°), hence the template maps are larger. (E) Because there is a topological isomorphism between the cortical surface, the left and right hemifields, and the model of retinotopy, the three representations have exact one-to-one correspondences. [file elife-40224-supp2.pdf]

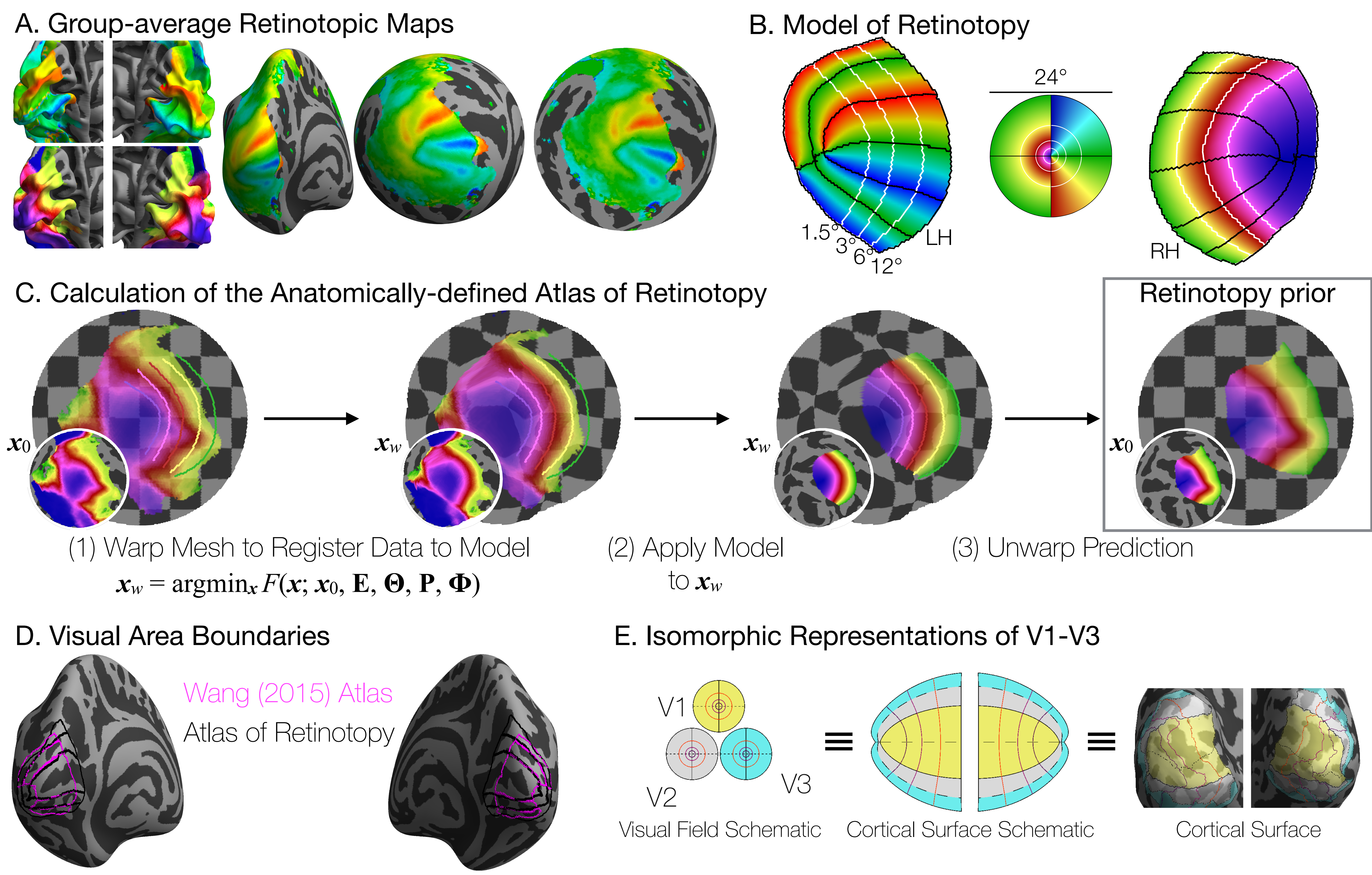

A. Group-average Retinotopic Maps
B. Model of Retinotopy
24°
1.5°
3°
6°
12°
LH
RH
C. Calculation of the Anatomically-defined Atlas of Retinotopy
Retinotopy prior
x_0
x_w
x_w
x_0
(1) Warp Mesh to Register Data to Model
$x_w = \mathrm{argmin}_x F(x; x_0, \mathbf{E}, \mathbf{\Theta}, \mathbf{P}, \mathbf{\Phi})$
(2) Apply Model
to x_w
(3) Unwarp Prediction
D. Visual Area Boundaries
Wang (2015) Atlas
Atlas of Retinotopy
E. Isomorphic Representations of V1-V3
V1
V2
V3
≡
≡
Visual Field Schematic
Cortical Surface Schematic
Cortical Surface
