# Supplementary figures and images for: Bayesian analysis of retinotopic maps
Source: eLife. 2018 Dec 6;7:e40224. doi: 10.7554/eLife.40224 (PMC6340702; doi:10.7554/eLife.40224)

A. 181 Subject Group-Average Retinotopic Maps

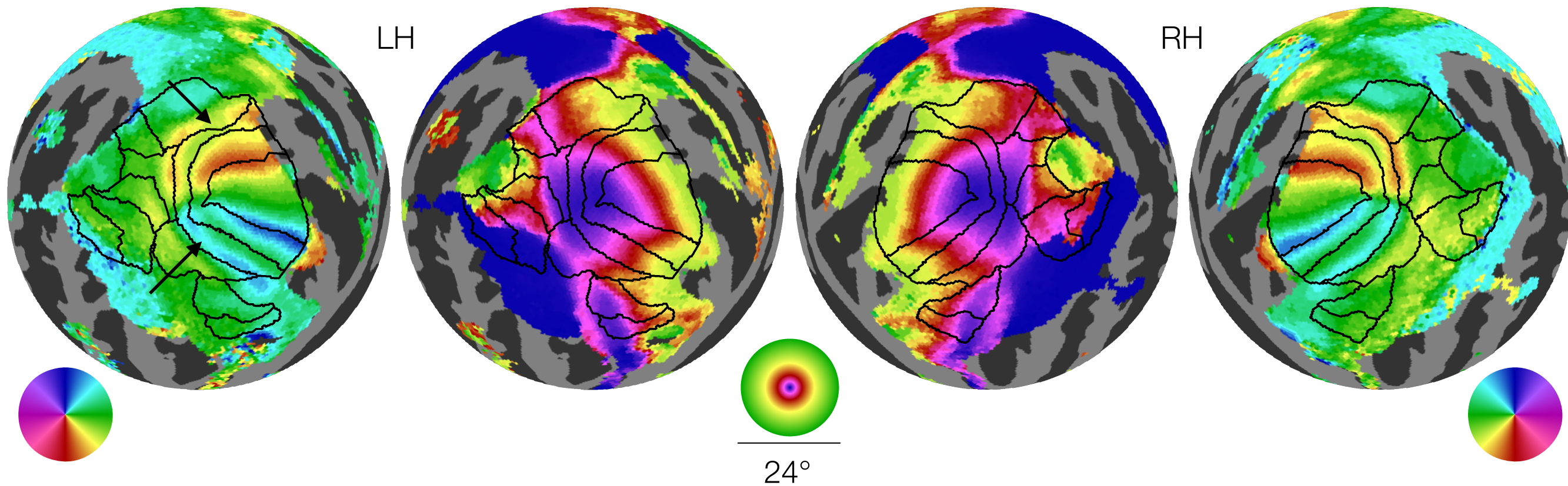

B. 181 Subject Group-Average Retinotopic Maps

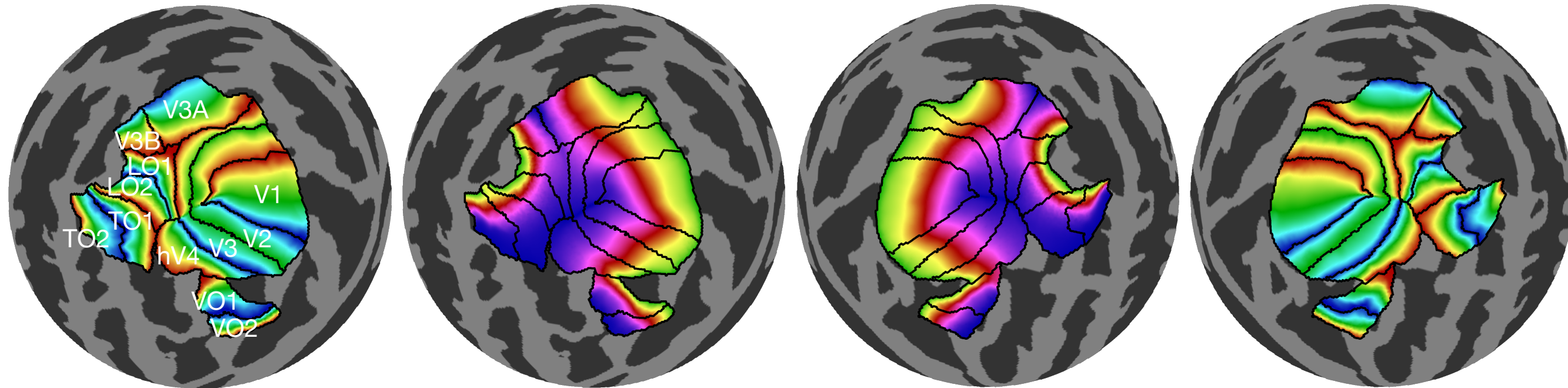

Supplement: Supplementary file 3. — (A) The 181-subject group-average retinotopic maps from the Human Connectome Project 7T Retinotopy Dataset are shown. These maps were used to construct the prior. Black arrows in the left-most plots indicate ‘notches’ of the V3 representation of the upper and lower vertical meridians that are absent in the group-average data. (B) The retinotopic prior is shown from 0 to 12° of eccentricity with boundary lines between areas. All 12 retinotopic areas included in the prior are shown. [file elife-40224-supp3.pdf]

Mean Warp Field

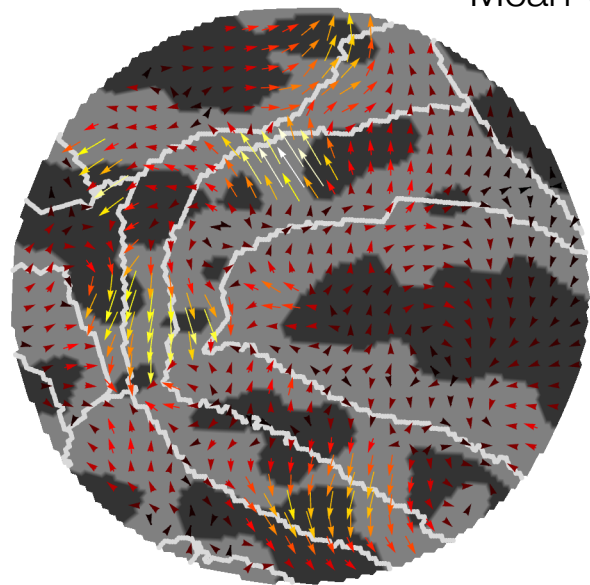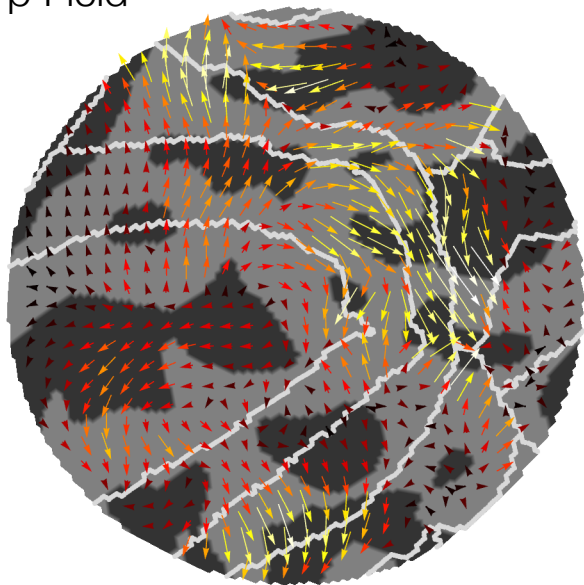

Principal Component 1

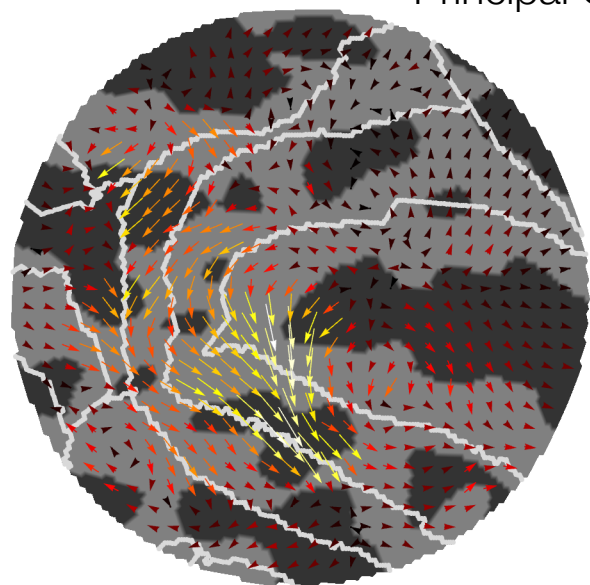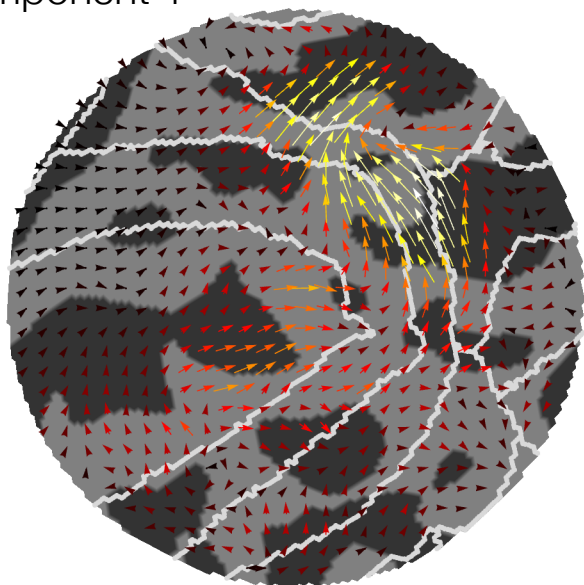

Principal Component 2

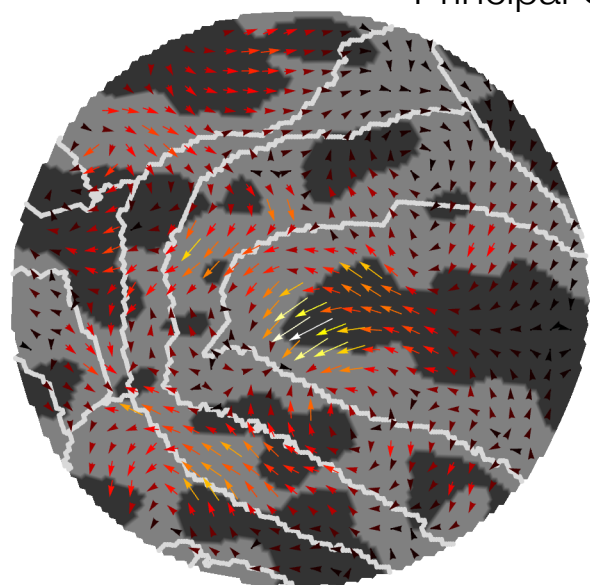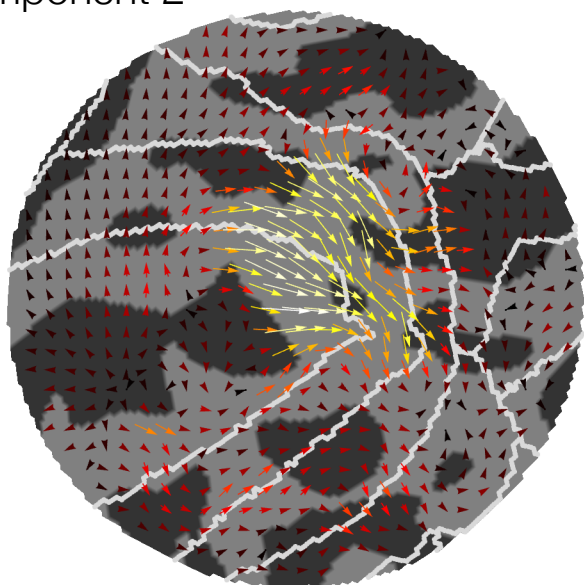

Principal Component 3

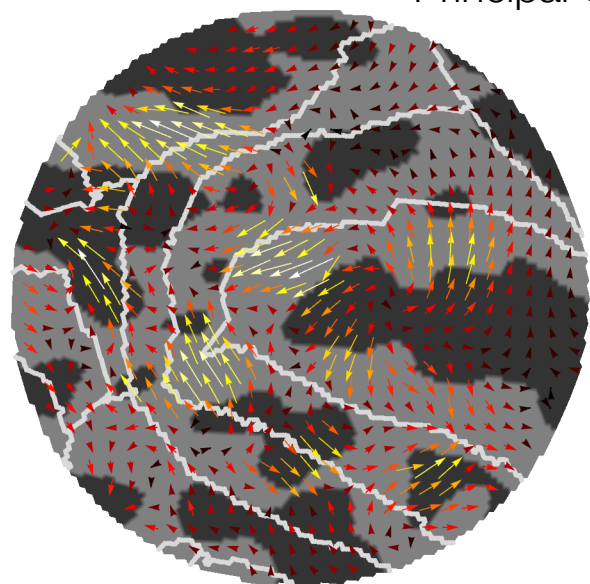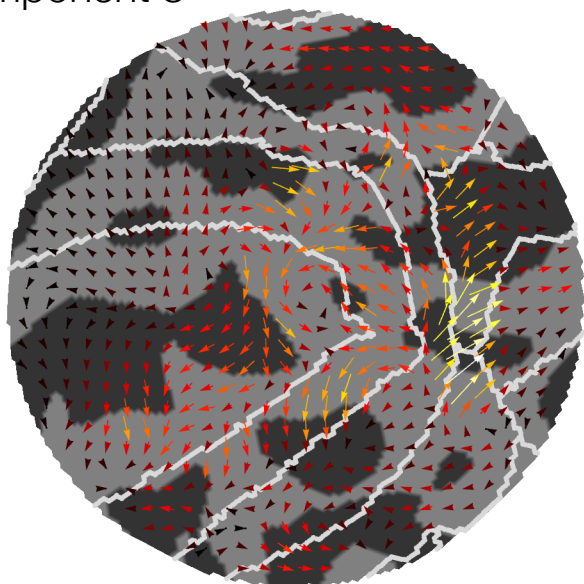

Supplement: Supplementary file 4. — The warp fields are calculated using the individual vertex deviations during the registration process (Figure 3C). The top row shows the mean vertex deformation across all subjects while the bottom three rows show the first three principal components of the deviations. The brightness of the arrows is based on their relative lengths. Note that because the top row shows the mean warp-field across subjects, the exact direction of the arrows is significant; however, in the bottom three rows, the principal component axes are shown, so the inversion of the arrows is equivalent to the plotted arrows. [file elife-40224-supp4.pdf]

A. HCP Subject 198653

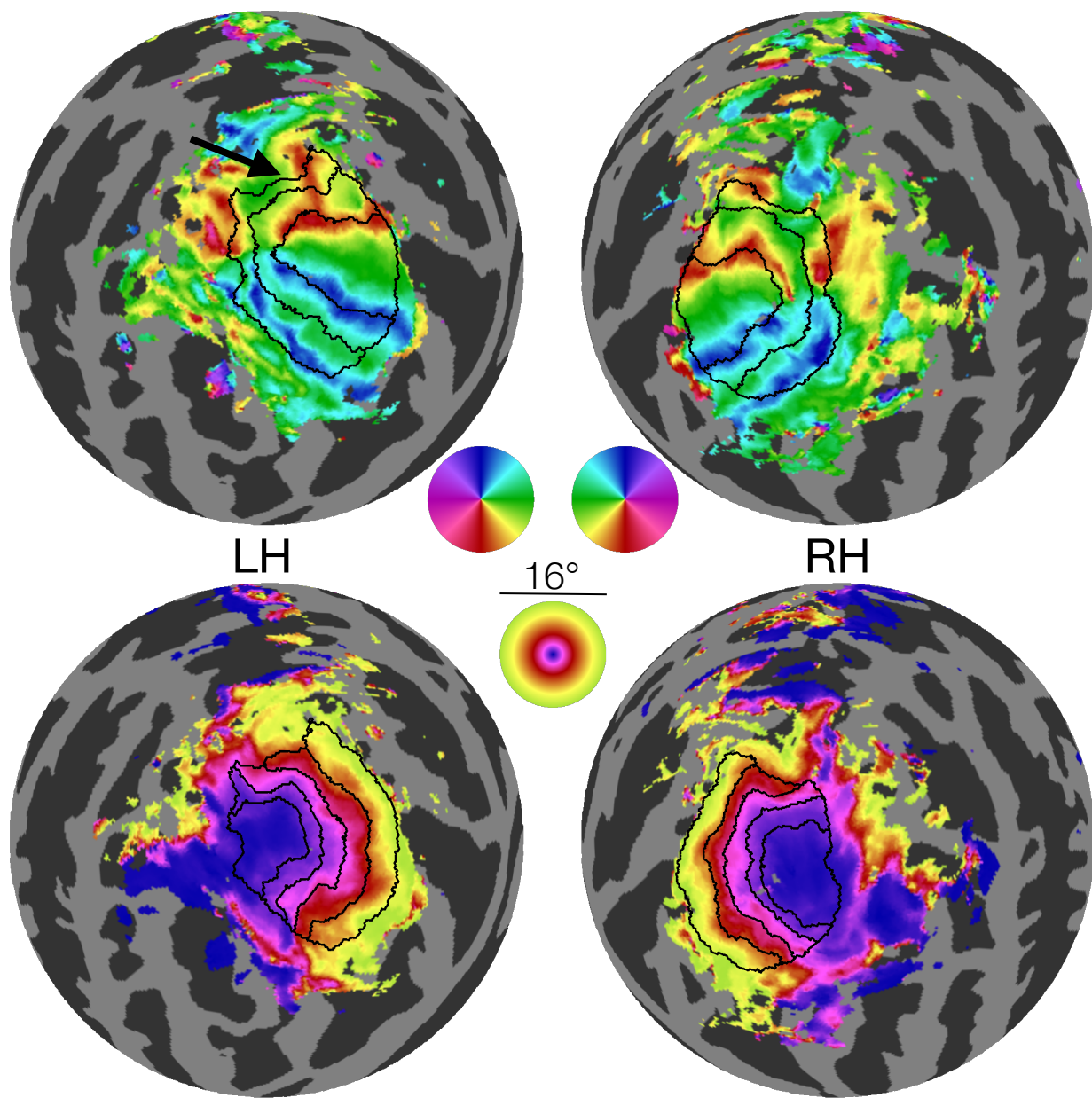

B. HCP Subject 644246

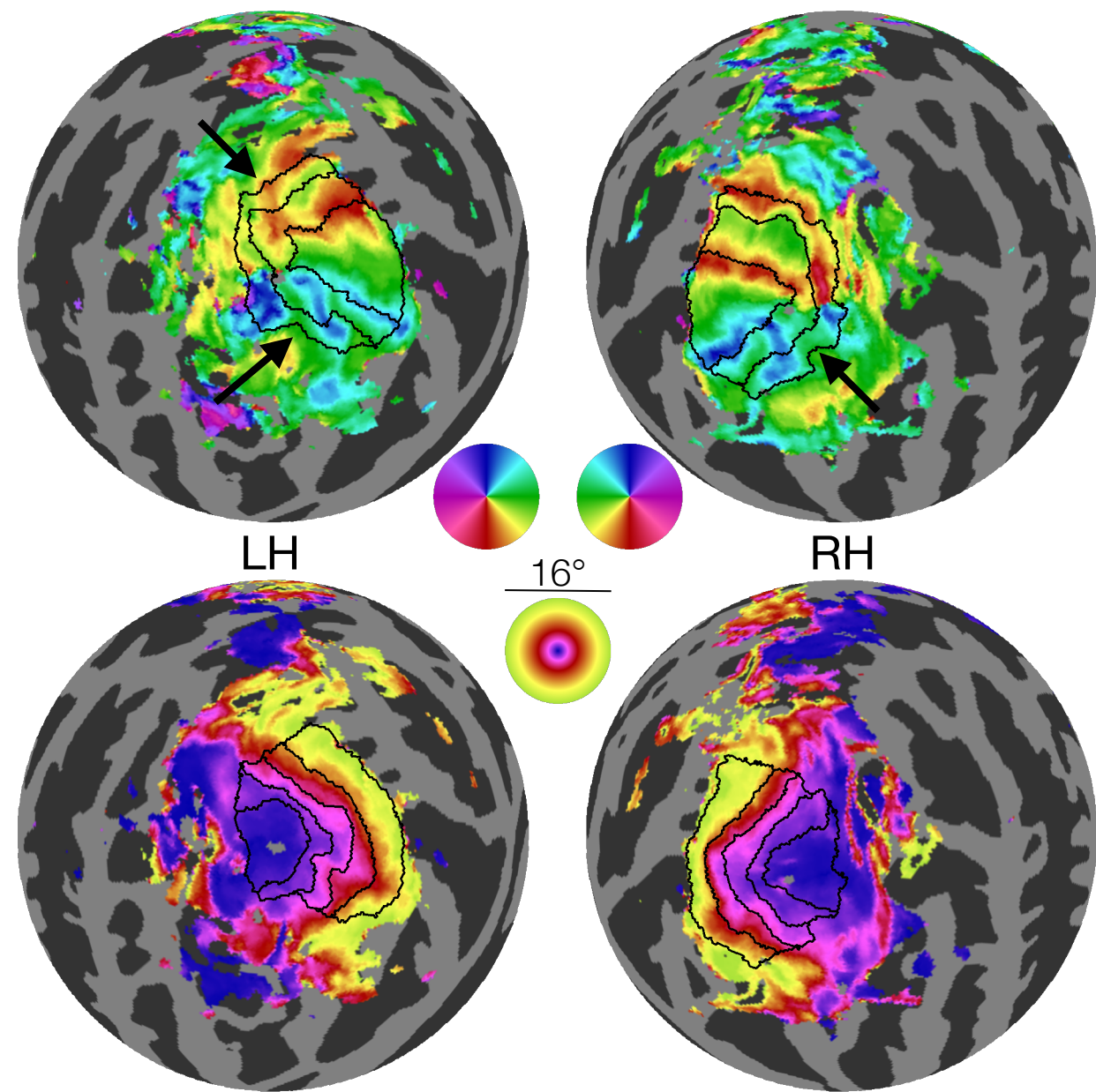

Supplement: Supplementary file 5. — (A) 198653 and (B) 644246 from the Human Connectome Project. These two subjects have unusual retinotopic organization in the polar angle maps of their left hemispheres (A) and on both hemispheres (B); this organization is not accounted for my our model of retinotopy and thus provides an example of how our Bayesian inference method performs when provided with atypical retinotopic maps. In the polar angle maps (top), black lines indicate V1/V2/V3 boundaries. In the eccentricity maps (bottom), black lines show the outer V3 boundaries and the 0.5°, 1°, 2°, 4° and 8° iso-eccentricity curves. Black arrows indicate the sites of atypical retinotopic organization. [file elife-40224-supp5.pdf]
